# Pediatric Acute Kidney Injury—The Time for Nihilism Is Over

**DOI:** 10.3389/fped.2020.00016

**Published:** 2020-01-31

**Authors:** Stuart L. Goldstein

**Affiliations:** Center for Acute Care Nephrology, Cincinnati Children's Hospital Medical Center, University of Cincinnati College of Medicine, Cincinnati, OH, United States

**Keywords:** acute kidney injury, children, renal replacement therapies, biomarkers, nihilism

## Abstract

Nihilism has been pervasive in the acute kidney injury field for decades, given that no studies, had been able to reduce AKI rates in hospitalized patients. Furthermore, children with AKI comprise an orphan population, where there is little incentive to develop diagnostics, therapeutics or devices specifically for them. The 3rd International Symposium on Acute Kidney Injury in Children, held in Cincinnati in October 2018, provided a platform to demonstrate the advancements in the diagnosis and treatment of children with, or at-risk for AKI, and also highlighted barriers to advancing care for this population. The progress made in the pediatric AKI since the 2nd International Symposium in 2016, highlighted the positive outcomes emanating from federal agency, private foundation and corporate sponsor investment in pediatric AKI. As a result, the time should be over for nihilism in the pediatric field.

## Introduction

Nihilism comes from the Latin *nihil*, or *nothing*. It is the belief that values are falsely invented. The term nihilism can also be used to describe the idea that life, or the world, has no distinct meaning or purpose. Nearly 20 years ago, Kellum and Angus wrote a landmark editorial reviewing the current state of acute renal failure (1). This editorial contained six foundational statements:

- A commonly held belief among intensivists and nephrologists is that patients die with, and not of, acute renal failure (ARF);
- Although this may seem a trivial distinction, its implications are far reaching;
- This raises the rather obvious but tricky questions of “why” and “what can we do to improve the situation”?
- Preventing the development of ARF in at-risk populations is an attractive but difficult goal;
- Well-powered studies have failed to demonstrate that drugs, such as low-dose dopamine or diuretics, can prevent onset or deterioration of renal function in the critically ill, and some studies have even suggested harm;
- The best advice to date is disappointingly empirical—avoid hypotension, dehydration, and exposure to nephrotoxins.

Implicit in these statements a heralding of a turning point from in the perspective from ignorance to nihilism, which in many cases, persists today. However, these statements also reflect the authors' hope that since patients are dying *from* and not just *with* their acute renal failure, it is incumbent upon clinician researchers in the field to not give into nihilism, but do something about it.

## Progress in Pediatric AKI

The 3rd International Symposium on Acute Kidney Injury in Children, held in Cincinnati in October 2018, provided a platform to demonstrate the advancements in the diagnosis and treatment of children with, or at-risk for acute kidney injury. Selected advancements are highlighted in this issue of *Frontiers in Pediatrics*. A tangible demonstration of the proverbial needle moving forward was a detailing of the fulfilled expectations from the end of the 2nd International Symposium held in 2016. At the end of the 2nd Symposium, I highlighted a number of areas I “hoped” we would be discussing in 2018:

1. AKI biomarker directed care algorithms;
2. Renal replacement therapy devices specifically designed for and/or targeted for uses in children with the plan for FDA clearance for use in children in the United States;
3. Dissemination of the successful single center nephrotoxic medication associated AKI program NINJA (2, 3), to multiple pediatric centers.

The progress toward each of these goals is detailed below.

### AKI Biomarker Directed Care Algorithms

Meersch and colleagues employed use of the cell-cycle arrest biomarkers, TIMP-2-IGBP7 (Nephrocheck™, Biomerieux, Inc.), to direct a care bundle in patients with an elevated biomarker product after cardiac surgery (4). Although this study was conducted in adults, the investigators demonstrated a significant reduction in AKI rates in the patients who received the bundle of care. Our team is currently conducting a prospective study in the pediatric ICU population to integrate a real time AKI risk assessment algorithm, the renal angina index (5–7), with urine AKI biomarker assessment (Neutrophil Gelatinase Associated Lipocalin, NGAL, BioPorto, Inc.), to guide fluid management and renal replacement therapy initiation (TAKING FOCUS 2, NCT03541785, 2P50 DK096418-06). Initial results were presented at the 3rd International Symposium showing patients who were RAI+ (with a score >8) and NGAL+ (with a concentration >150 ng/ml), comprised an overwhelmingly majority of patients who developed >10% fluid overload and required renal replacement therapy. The importance of using an AKI biomarker in these studies is to enrich the sample of patients to include only those who would be truly at increased risk for AKI development. These studies should serve as a trial design template for future interventional trials aimed at preventing AKI or mitigating its effects.

### Renal Replacement Therapy Devices Specifically Designed for and/or Targeted for Uses in Children With the Plan for FDA Clearance for Use in Children in the United States

Currently, three different devices have completed studies and/or have applications in process with the FDA for a pediatric indication. The HF20™ CRRT circuit (Baxter Healthcare, McGaw Park, IL), would represent that small dedicated CRRT circuit available in the US. It has been used in countries outside of the United States for over 10 years (8). A five center US pediatric consortium completed a prospective study with the HF20™ in 2018 with the aim of FDA clearance (NCT02561247). The Cardiorenal Pediatric Emergency Dialysis Machine (CARPEDIEM™, Medtronic, Inc., Mirandola Italy) is a CRRT machine designed specifically for neonates with AKI and is available in Europe (9, 10). The FDA is currently reviewing a submission for the CARPEDIEM™ for clearance for use in the United States. The Selective Cytopheretic Device (SCD, Seastar, Inc., San Diego, CA), has been demonstrated to improve outcomes in adult patients with AKI receiving CRRT, where the circuit ionized calcium is maintained at <0.4 mmol/L (11). The SCD is use in tandem with CRRT and its mechanistic effect is by immune modulation. Currently, a 5 center US consortium is prospectively evaluating the SCD in pediatric patients (NCT02820350, R01FD005092). In addition, since the time of the 2018 Symposium, a multicenter retrospective US study has detailed the use and associated outcomes of an ultrafiltration device (Aquadex™, CHF Solutions, Inc, Minneapolis, MN) to support children with AKI and/or fluid overload (12). Thus, these studies clearly demonstrate that despite significant challenges, devices are being made specifically for children, or successfully adapted for them. Hopefully, the devices under consideration at FDA will be cleared and made available in the US.

### Dissemination of the Successful Single Center Nephrotoxic Medication Associated AKI Program NINJA, to Multiple Pediatric Centers

Nephrotoxic medication exposure represents one of the most common causes of AKI in hospitalized children. Our center realized a 38% reduction in nephrotoxic medication exposure and a 62% reduction in associated AKI after implementation of the Nephrotoxic Injury Negated by Just in time Action (NINJA) program (3). NINJA identifies patients in near real time who are exposed to three or more nephrotoxic medications on the same day or receiving an IV aminoglycoside or IV vancomycin for three or more days. Exposed patients are then recommended to have a daily serum creatinine to assess for AKI development systematically. A nine center collaborative recently completed a 3 year implementation of NINJA (1R18HS023763-01) to determine if NINJA could be successfully disseminated to these centers and to ascertain the contextual factors that accelerated or hindered successful implementation. Preliminary data presented at the 3rd International Symposium showed a 23.8% reduction in AKI rates across the collaborative. Other data from this effort assessed projected health care cost reductions associated with NINJA, and various AKI rates in different service lines in the NINJA collaborative.

## Conclusions

While the nihilistic perspective Kellum and Angus were concerned about nearly 20 years may still persist in some circles, the advancements in AKI clinical care, research and investment in pediatric AKI research and devices, suggests the tide may be finally turning. The progress made in the past 2 years has been especially dramatic, as highlighted in the pages of this Golden Research Topic volume. With persistence and determination, a brighter future should be realized to improve outcomes for children with, or at-risk for AKI. In the not too distant future, the time for nihilism will be over.

## Disclosure

Industry sponsors mentioned in this manuscript, Baxter Healthcare, Medtronic, BioPorto and CHF Solutions provided unrestricted educational grants for the 3rd International Symposium on AKI in Children. Dr. Goldstein serves as a consultant for all of the industry sponsors as well.

## Author Contributions

The author confirms being the sole contributor of this work and has approved it for publication.

### Conflict of Interest

Industry sponsors mentioned in this manuscript, Baxter Healthcare, Medtronic, BioPorto, and CHF Solutions provided unrestricted educational grants for the 3rd International Symposium on AKI in Children. SG serves as a consultant for all of the industry sponsors as well.
